# Structural Alteration in Dermal Vessels and Collagen Bundles following Exposure of Skin Wound to Zeolite–Bentonite Compound

**DOI:** 10.1155/2016/5843459

**Published:** 2016-12-28

**Authors:** Shahram Paydar, Ali Noorafshan, Behnam Dalfardi, Shahram Jahanabadi, Seyed Mohammad Javad Mortazavi, Seyedeh-Saeedeh Yahyavi, Hadi Khoshmohabat

**Affiliations:** ^1^Trauma Research Center, Shahid Rajaee (Emtiaz) Trauma Hospital, Shiraz University of Medical Sciences, Shiraz, Iran; ^2^Department of General Surgery, School of Medicine, Shiraz University of Medical Sciences, Shiraz, Iran; ^3^Histomorphometry and Stereology Research Centre, Shiraz University of Medical Sciences, Shiraz, Iran; ^4^Student Research Committee, Shiraz University of Medical Sciences, Shiraz, Iran; ^5^Department of Internal Medicine, Shiraz University of Medical Sciences, Shiraz, Iran; ^6^International Branch, Shiraz University of Medical Sciences, Shiraz, Iran; ^7^Medical Physics Department, School of Medicine, Shiraz University of Medical Sciences, Shiraz, Iran; ^8^Ionizing and Non-Ionizing Radiation Protection Research Center (INIRPRC), Shiraz University of Medical Sciences, Shiraz, Iran; ^9^Trauma Research Center, Baqiyatallah University of Medical Sciences, Tehran, Iran

## Abstract

*Background*. This study examines the impact of one-time direct application of haemostatic agent zeolite–bentonite powder to wounded skin on the healing process in rats.* Materials and Methods*. 24 male Sprague-Dawley rats were randomly allocated into two groups (*n* = 12): (1) the rats whose wounds were washed only with sterile normal saline (NS-treated) and (2) those treated with zeolite–bentonite compound (ZEO-treated). The wound was circular, full-thickness, and 2 cm in diameter. At the end of the 12th day, six animals from each group were randomly selected and terminated. The remaining rats were terminated after 21 days. Just after scarification, skin samples were excised and sent for stereological evaluation.* Results*. The results showed a significant difference between the two groups regarding the length density of the blood vessels and diameter of the large and small vessels on the 12th day after the wound was inflicted. Besides, volume density of both the dermis and collagen bundles was reduced by 25% in the ZEO-treated rats in comparison to the NS-treated animals after 21 days.* Conclusions*. One-time topical usage of zeolite–bentonite haemostatic powder on an animal skin wound might negatively affect the healing process through vasoconstriction and inhibition of neoangiogenesis.

## 1. Introduction

High bleeding still accounts for up to 40% of preventable deaths following traumatic injuries [1]. Haemostatic agents can considerably contribute to stopping such uncontrolled haemorrhage in trauma patients and reduce the possibility of its undesirable consequences [1, 2]. However, in spite of their usefulness in emergency cases, haemostatic materials are not available in all parts of the world. Moreover, an ideal haemostat has not been produced yet. Hence, improvement of the available haemostatic agents and production of new ones have remained in focus [2, 3].

One haemostatic agent that was recently produced in Iran (called CoolClot) is a compound mainly composed of zeolite (one third of the weight) and bentonite (two thirds of the weight) clays, two minerals which are widely available in the country [3, 4]. This recently introduced product was originally made in powder form and has been proven to control life-threatening arterial bleeding. Another advantage of this product is that it does not have the potential burn effects caused by other zeolite-based haemostatic agents [3–5].

The criteria for the idealness of haemostatic agents include the ability to stop haemorrhage from making large arteries or veins actively bleed within two minutes of application; being ready to use with no requirement for on-site preparation; the ability to deliver and act through a pool of blood; being risk-free; causing no further tissue injury; having no negative impact on the wound; having no risk of viral disease transmission; being easy to use for the casualty, medical staff, and nonmedical first responders; being lightweight and durable; having wide temperature storage capabilities (ideally −10 to +55°C); having a minimum of two years shelf-life; and being inexpensive [1–3]. Considering these criteria, it seems reasonable to assess different aspects of any newly introduced haemostatic agent, particularly its impact on wound healing and tissue safety.

According to the aforementioned criteria, an ideal haemostatic material should have no negative impact on the fresh wound (site of topical application) and its healing process [3]. A method that is used in dermatological research, particularly for assessment of wound healing, is stereology. This technique is defined as a set of mathematical and statistical tools that estimate three-dimensional features of objects from their regular two-dimensional sections and provide the examiner with a better understanding of the tissue morphology [6–9].

This study aims to stereologically evaluate the impact of one-time topical application of zeolite–bentonite compound on the skin wound healing process in an animal model and assess its tissue safety.

## 2. Materials and Methods

### 2.1. Animals and Wound Creation

To conduct this research, according to a previously used method [3], 24 healthy adult male Sprague-Dawley rats (mean weight: 230 g) were chosen and randomly divided into two groups (*n* = 12): (1) normal saline-treated group (NS-treated) and (2) zeolite–bentonite compound-treated group (ZEO-treated). The animals were housed in temperature- and humidity-controlled rooms with 12-hour light/dark photoperiods and had free access to similar amounts of standard food (provided from the Centre of Comparative and Experimental Medicine, Shiraz University of Medical Sciences, Fars province, Shiraz, Iran) and water. The rats were adapted to their environment 10 days prior to the start of the study. The research protocol was approved by the Animal Ethics Committee of Shiraz University of Medical Sciences, Shiraz, Iran.

To begin the experiment under general anaesthesia (through the intramuscular injection of ketamine [50 mg per kg; Alfasan International, Woerden, The Netherlands] and xylazine [10 mg per kg; Alfasan International]) in a clean but not sterile condition, a circular full thickness cutaneous wound with 2 cm diameter was generated on the dorsum of each animal using forceps and scissors. Just after wound creation, a single dose of zeolite–bentonite powder (in dry form; with a mean weight of 8 gr) was applied on the skin wound of the rats in the ZEO-treated group. The powder was spread as uniformly as possible on the wound site using a sterile applicator stick. The cutaneous wounds of the animals in the NS-treated group were only washed with sterile normal saline. From the second day of the research, the skin wounds of both groups of animals were washed daily with normal saline. The wounds were left completely open during the study time span.

At the end of the 12th day, six animals per group were randomly selected and terminated with a high dose of inhaled ether. After 21 days, the remaining animals were also terminated by a similar method. Just after scarification, full thickness skin samples were excised from the site of the wounded skin. The samples were fixed in buffered formaldehyde and sent for further processing.

### 2.2. Stereological Analysis

We used stereological analysis to examine tissue samples [10]. The samples were sectioned into 0.5 × 0.5 mm^2^ and nine to 10 pieces were sampled in a systematic uniform random pattern. Isotropic uniform random sectioning is necessary for the estimation of the vessels' length. Therefore, the samples were embedded in a cylindrical block and sectioned using a microtome after choosing random orientation according to the orientator method. In this way, four-micrometer sections were obtained and stained with Heidenhain's AZAN trichrome stain.

Microscopic analyses of the skin were performed using a video-microscopy system composed of a microscope (Nikon E-200, Tokyo, Japan) linked to a digital camera and a flat monitor. The volume densities (the fraction of the tissue which was occupied by the favoured structure)—of parts including epidermis, dermis, hypodermis, collagen bundles, and vessels—were estimated using the point-counting method. Briefly, a point grid was overlaid on the images of the skin and the density was obtained using the following formula:

(1) $$\begin{array}{c} V_{v}\left(\;structure,reference\;\right)=\frac{\Sigma P\left(structure\right)}{\Sigma P\left(reference\right)}, \end{array}$$

where “Σ*P*(structure)” and “Σ*P*(reference)” were the total points hitting the favoured structure and the whole skin sections, respectively.

The length density (*L*_*v*_, the length of the vessels in the unit volume of the dermis and hypodermis) and the mean diameter of a vessel were estimated using a counting frame at a final magnification of 1380x and 130x to differentiate the capillaries (up to 10 *µ*m) and larger vessels (more than 10.1 micrometer), respectively.

The following formula was used to estimate the length density:

(2) $$\begin{array}{c} L_{v}=2\times \frac{\Sigma Q}{\left[\left(a/f\right)\times \Sigma P\right]}, \end{array}$$

where “Σ*Q*”, “(*a*/*f*)”, and “Σ*P*” were the total profiles of the vessels, area per counting frame and the total hitting of central point of the frame with the reference tissue.

### 2.3. Statistical Analysis

Data analysis was performed using the IBM SPSS Statistics software, v. 16 (Chicago, USA). Further, Mann-Whitney *U*-test was used to analyse the histological data. *P* < 0.05 was considered statistically significant.

## 3. Results

**Figure 1:**
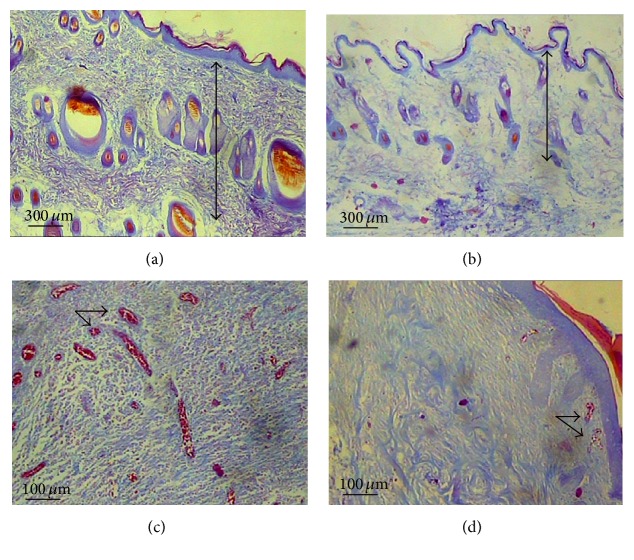
The photomicrograph of the rat skin stained with Heidenhain's AZAN trichrome. (a) and (b) images show the wounded skin of the rats 21 days after treatment with NS and ZEO, respectively. The two-head arrows indicate reduction in the dermis size of the ZEO-treated animals. (c) and (d) images display the dermis of the rats 21 days after treatment with normal saline and ZEO, respectively. The arrows indicate reduction in the vessels' density and diameter in the ZEO-exposed rats.

The results showed no significant difference in NS- and ZEO-treated groups regarding the volume density of the epidermis, dermis, and hypodermis of the wounded skin after 12 days (Table 1) (Figure 1). However, the length density of the vessels was reduced by 31% in the ZEO-treated group after this period. The diameters of the large and small vessels (capillaries) were also, respectively, reduced by 38% and 16% in the ZEO-treated rats in comparison to the other group during 12 days. Additionally, volume density of both the dermis and collagen bundles was reduced by 25% in the ZEO-treated rats in comparison to the NS-treated animals after 21 days. Nevertheless, no significant changes were seen in the epidermis and hypodermis of the animals' skin samples in both groups at that time. Length density of the vessels was reduced by 46% in the experimental group. The diameters of the large vessels and capillaries was also reduced, by 39% and 16%, respectively, in the ZEO-treated rats compared to the controlled ones after 21 days. Reduction of the length density and diameter indicates angioinhibitory and vasoconstrictive actions in 12 and 21 days post-wounding.

## 4. Discussion

This work assessed the impact of one-time direct application of zeolite–bentonite powder to a wounded skin tissue on its healing process. Our findings showed that this compound could affect the healing process through angioinhibitory and vasoconstrictive properties. In addition, this study indicated that application of zeolite–bentonite compound could reduce the volume density of collagen bundles in the healed tissue.

Because of some previously poor experiences regarding the use of haemostatic products, their safety for human tissues remains a constant concern [13]. For instance, QuikClot (Z-Medica, Wallingford, CT, USA) could cause significant exothermic reaction, thermal tissue injury, and necrosis after topical usage (it is claimed that this side effect is considerably resolved in the later generation of this agent called QuikClot ACS+™) [11]. Dry Fibrin Sealant Dressing (DFSD, American Red Cross Holland Laboratory, Rockville, MD) could also transmit viral diseases (particularly hepatitis and human immunodeficiency virus) [12]. Hence, this issue is a detriment to the idealness of haemostatic agents as they should be risk-free and cause no further tissue injury [1–3]. Therefore, assessment of the safety of any newly introduced haemostatic material is necessary. Zeolite–bentonite powder, as a product whose impact on wounded tissues has not been completely examined, is no exception.

Previously, Khoshmohabat et al. performed an experiment to histopathologically evaluate the effects of one-time topical administration of a zeolite–bentonite composition called CoolClot, on the skin wound healing process [3]. In that study, three main and overlapping phases of cutaneous wound healing—including inflammation, proliferation, and maturation—were examined using the scoring system introduced by Abramov and his colleagues (Table 2) [3, 13, 14]. It is noteworthy that Abramov's scoring system was developed to make qualitative histopathological data available for secondary statistical analysis. According to the results of that study, one-time topical usage of CoolClot had no significant negative impact on the wound healing process, neither histopathologic nor macroscopic (photographic) [3]. However, our study, having quantitatively evaluated the structural changes of skin using stereological methods, revealed different results from those of the previous histopathologic research. According to our results, one-time topical application of zeolite–bentonite compound could lead to angioinhibitory and vasoconstrictive features that remained even towards the end of the healing process.

Angiogenesis or neovascularization is a critical event occurring during wound repair [15]. Furthermore, newly established blood supply is essential to provide the metabolic demands of the healing tissues. In addition, vasodilatation is a necessary component for angiogenesis. It has been shown that various cell types, such as epidermal cells, macrophages, fibroblasts, and vascular endothelial cells, can stimulate neovascularization by the production of different factors, including transforming growth factor ß (TGFß), nitric oxide (NO), vascular endothelial growth factor (VEGF), and basic fibroblast growth factor (bFGF) [14–17]. Our stereological study indicated that zeolite–bentonite powder could negatively affect angiogenesis and its associated vasodilation. Consequently, it can have a negative impact on the healing process. However, it remains questionable why and by which mechanism zeolite–bentonite compound could affect the aforementioned events.

Another important point is that the current results of the study show that the volume density of the collagen bundles was significantly less in the healing tissues of the ZEO-treated rats compared to the NS-treated group on Day 21. It is evident that collagen deposition by fibroblasts is essential for the healing process and provides tissue strength, integrity, and structure. However, excessive amounts of collagen deposition will increase the probability of scar formation [16]. According to our findings, it could be claimed that the possibility of scar formation was less in the ZEO-treated animals in comparison to the other group. Yet, it seems necessary to objectively measure the healed tissue strength to opine about the impact of reduction in volume density of collagen on tissue strength.

This work had some limitations that should be considered while interpreting the findings. First, the present study examined the impact of the topical application of zeolite–bentonite compound on a skin wound model using stereological techniques. Nonetheless, we did not photographically compare wound surface area between NS- and ZEO-treated groups during the study period. In fact, although our findings showed that the zeolite–bentonite powder could have angioinhibitory and vasoconstrictive features, these effects might result in no significant macroscopic difference between the NS- and ZEO-treated groups regarding the wound surface area. This is similar to the result obtained in the experiment by Khoshmohabat et al. Another limitation of the current work was not comparing the impact of zeolite–bentonite powder on wound healing to that of an FDA-approved haemostatic agent to which we had no access. Because of such inaccessibility, we washed the wounds of the control group (NS-treated animals) using only sterile normal saline. Third, we assessed the skin biopsies taken in the middle and late postwounding periods, although it would have been beneficial to examine the skin biopsies of other groups of animals, stereologically, early on (e.g., on Day 4 or 5). However, our limitation in providing male rats was an obstacle to this. Due to the impact of sex hormones on the wound healing process and fluctuation of their levels during the reproductive cycle in female rats, we could not use them in our work.

In conclusion, according to this stereological study, one-time topical usage of zeolite–bentonite haemostatic powder on an animal skin wound could negatively affect the healing process by inhibition of neovascularization and vasoconstriction. In addition, due to the reduction of volume density of collagen bundles after application of zeolite–bentonite compound, the healing tissues had a lesser possibility of scar formation.

## Figures and Tables

**Table 1:** The mean and standard deviation of the animal weight, volume density (mm^3^/mm^3^, ×100) of the different layers of the skin, collagen bundle, length density (mm/mm^3^), and diameter (*µ*m) of the vessels, 12 and 21 days after treating the rats with NS and ZEO. (*n* = 6).

Groups	Volume density				Length density	Diameter	
	Epidermis	Dermis	Hypodermis	Collagen	Vessels	>10 *µ*m	<10 *µ*m
NS-treated (12)	2.6 ± 0.6	17.6 ± 6.4	79.6 ± 6.7	45.1 ± 6.0	6.8 ± 0.9	37.5 ± 10.1	6.1 ± 0.7
ZEO-treated (12)	2.1 ± 0.8	20.0 ± 0.4	77.8 ± 4.5	45.9 ± 9.9	4.7 ± 1.2^*∗*^	23.3 ± 7.8^*∗*^	5.0 ± 0.7^*∗*^
NS-treated (21)	3.6 ± 0.7	49.8 ± 8.9	46.4 ± 9.1	45.6 ± 9.3	5.0 ± 2.9	37.3 ± 5.7	6.0 ± 0.2
ZEO-treated (21)	3.1 ± 1.3	37.5 ± 7.7^*∗*^	59.3 ± 7.5	34.2 ± 6.5^*∗*^	2.7 ± 1.2^*∗*^	22.9 ± 2.7^*∗*^	5.0 ± 0.3^*∗*^

^*∗*^ *P* < 0.03, ZEO-treated *versus* NS-treated animals.

**Table 2:** The Abramov's histological scoring system for wound repair.

Parameter	Score			
	0	1	2	3
Acute inflammation	None	Scant	Moderate	Abundant
Chronic inflammation	None	Scant	Moderate	Abundant
Amount of granulation tissue	None	Scant	Moderate	Abundant
Granulation tissue maturation	Immature	Mild maturation	Moderate maturation	Fully matured
Collagen deposition	None	Scant	Moderate	Abundant
Reepithelialization	None	Partial	Complete but immature or thin	Complete and mature
Neovascularization	None	Up to five vessels per HPF^*∗*^	6 to 10 vessels per HPF	More than 10 vessels per HPF

^*∗*^High power field.
